# An Exploratory Pilot Study of Genetic Marker for IgE-Mediated Allergic Diseases with Expressions of FcεR1α and Cε

**DOI:** 10.3390/ijms16059504

**Published:** 2015-04-27

**Authors:** En-Chih Liao, Ching-Yun Chang, Chia-Wei Hsieh, Sheng-Jie Yu, Sui-Chu Yin, Jaw-Ji Tsai

**Affiliations:** 1 Center for Translational Medicine, Department of Medical Research, Taichung Veterans General Hospital, Taichung 40705, Taiwan; E-Mails: en65@vghtc.gov.tw (E.-C.L.); scyin@vghtc.gov.tw (S.-C.Y.); 2 Department of Bio-Industry Technology, Da Yeh University, Changhua 51591, Taiwan; 3 Department of Medical Technology, Jen Ten College of Medicine, Nursing and Management, Miaoli 35664, Taiwan; 4 Division of Allergy, Immunology & Rheumatology, Department of Internal Medicine, Taichung Veterans General Hospital, Taichung 40705, Taiwan; E-Mails: yun0922.tw@yahoo.com.tw (C.-Y.C.); chiaweih@gmail.com (C.-W.H.); 5 College of Life Sciences, National Chung Hsing University, Taichung 40227, Taiwan; E-Mail: jim0929@msn.com; 6 Institute of Clinical Medicine, School of Medicine, National Yang Ming University, Taipei 11221, Taiwan

**Keywords:** FcεR1α promoter region, FcεR1α mRNA expression, single nucleotide polymorphism (SNP), genetic markers, allergic diseases

## Abstract

The high affinity immunoglobulin E (IgE) receptor-FcεR1 is mainly expressed on the surface of effector cells. Cross-linking of IgE Abs bound to FcεR1 by multi-valent antigens can induce the activation of these cells and the secretion of inflammatory mediators. Since FcεR1 plays a central role in the induction and maintenance of allergic responses, this study aimed to investigate the association of FcεR1 with the allergic phenotype of Cε expression and cytokine and histamine release from peripheral leukocytes. Peripheral leukocytes from 67 allergic and 50 non-allergic subjects were used for genotyping analysis. Peripheral mononuclear cells (PBMCs) were used for Cε expression and ELISpot analysis, while polymorphonuclear cells (PMNs) were used for histamine release. The association between genotype polymorphism of the FcεR1α promoter region (rs2427827 and rs2251746) and allergic features of Cε expression and histamine were analyzed, and their effects on leukocytes function were compared with wild type. The genotype polymorphisms of FcεR1α promoter region with CT and TT in rs2427827 and TC in rs2251746 were significantly higher in allergic patients than in non-allergic controls. Patients with single nucleotide polymorphism (SNP) of FcεR1α promoter region had high levels of total IgE, mite-specific Der p 2 (Group 2 allergen of *Dermatophagoides pteronyssinus*)-specific IgE and IgE secretion B cells. The mRNA expression of FcεR1α was significantly increased after Der p2 stimulation in PBMCs with SNPs of the FcεR1α promoter region. Despite the increased Cε mRNA expression in PBMCs and histamine release from PMNs and the up-regulated mRNA expression of interleukin (IL)-6 and IL-8 secretions after Der p2 stimulation, there was no statistically significant difference between SNPs of the FcεR1α promoter region and the wild type. SNPs of FcεR1α promoter region were associated with IgE expression, IgE producing B cells, and increased Der p2-induced FcεR1α mRNA expression. These SNPs may be used as a disease marker for IgE-mediated allergic inflammation caused by *Dermatophagoides pteronyssinus*.

## 1. Introduction

Immunoglobulin E (IgE) is most frequently recognized for its role in type-I hypersensitivity reaction. During the IgE-mediated response, IgE binds with a high-affinity IgE receptor consisting of a tetramer of a ligand-binding α chain (FcεR1α), a signal-augmenting β, and a signal-transducing γ chain dimer. The receptor is abundantly expressed on the surface of mast cells and basophils, and is thought to be involved in allergic inflammation of the asthmatic airway. Because high total serum IgE levels are closely correlated with the clinical expression and severity of asthma and allergy, IgE is thought to play a key role in the pathogenesis of allergic diseases [1,2]. The regulation of serum IgE production is largely influenced by familial determinants [3,4]. Genetic susceptibility to IgE-responsiveness is likely to be caused by a pattern of polymorphisms in multiple genes regulating immunologic responses [5]. Recently, Chen *et al.* found that a common variant in FcεR1α was associated with total serum IgE levels in cord blood or blood samples from birth up to the first six years of life, and this was independent of environmental endotoxin exposure from house dust samples [6].

The FcεR1, also known as high-affinity IgE receptor, is the high affinity receptor for the Fc region of IgE and is a tetrameric receptor complex consisting of one α (FcεR1α-antibody binding site), one β (FcεR1β-which amplifies the downstream signal), and two disulfide bridge connected γ chains (FcεRIγ-the site where the downstream signal initiates). The binding itself of monomeric IgE to FcεR1 can promote the survival of mast cells without cross-linking of the receptor [7,8], suggesting that an increase in the FcεR1α-chain on the cell surface accelerates the IgE-mediated allergic reaction. Further, the involvement of the α-chain in FcεR1-mediated allergic reaction has been definitively proven by the absence of any allergic reaction in the α-chain-deficient mice. A genetic linkage to atopic dermatitis has been recently assigned to human chromosome 1q21, which is very close to the chromosomal locus where FcεR1α is mapped. However, a possible polymorphism in FcεR1α, which encodes the α chain of the high affinity receptor for IgE may be associated with the functional variants of IgE expression in allergic diseases [9]. A recent fine-mapping study confirmed the IgE-associated loci 1q23 (*FCER1A*) using 1000 Genomes Project datasets and identified its concomitant contribution to IgE regulation [10].

Although no interaction between the single nucleotide polymorphism (SNP) in the FcεR1α and environmental exposures including endotoxin was observed, the SNP in the FcεR1α exhibits strong effects on total serum IgE levels [6]. It suggests that the functional promoter variant in FcεR1α as a good candidate for contributing to total serum IgE. The genetic polymorphism of the FcεR1 promoter region plays an important role in the pathogenesis of IgE-mediated allergic inflammation. The α-chain genes of FcεR1 have been demonstrated to be cooperatively transactivated by the transcription factors GATA-1 and PU.1. Recently, studies on SNPs in the FcεR1α promoter regions affected by transcription factors have revealed that these genes are probable candidates for allergy-related genes. Immunoglobulin class switching, also known as class-switch recombination (CSR), is a biological mechanism for a B cell’s production of immunoglobulin from the isotype IgM to isotype IgG. The CSR replaces initially the Ig heavy chain constant region (C_H_) gene to be expressed from Cμ(IgM) to other C_H_ genes, resulting in switch of the Ig isotype from IgM to either IgG, IgA or IgE. Stimulation of B-cells with anti-CD40 plus IL-4 induced CSR from Cμ to Cε (IgE), which contributes to the pathogenesis of allergic diseases. This study investigated the association of genetic polymorphisms in the FcεR1α promoter region with the clinical features of allergy, and analyzed its genetic effect on the mRNA expression of FcεR1α and Cε.

## 2. Results

### 2.1. Association of Single Nucleotide Polymorphisms (SNPs) of the FcεR1α Promoter Region with Allergic Clinical Features

The association of genetic polymorphisms of the FcεR1α promoter region with clinical features of allergy was analyzed in patients with allergy. Two different genotypes of FcεR1α promoter regions, rs2427827 (−344) and rs2251746 (−95), were analyzed (Table 1). The genotypes rs2427827 (−344, C>T) and rs2251746 (−95, T>C) were associated with high levels of total IgE, specific mite-IgE, specific Der p2-IgE, and numbers of B cells that produced IgE after Der p2 stimulation. The comparison of clinical characteristics between allergic and healthy subjects was shown in supplementary Table S1. There were significant differences of total IgE, mite-IgE and Der p 2-IgE between allergic and healthy subjects.

### 2.2. Genotype and Allele Frequency of the FcεR1α Promoter Region in Allergic Subjects

Comparisons of the genotype and frequency of FcεR1α polymorphism in the promoter region between allergic patients and healthy controls revealed that the genotype of the FcεR1α promoter region with CT and TT in rs2427827 (−344) and TC in rs2251746 (−95). The genotypes of the rs2427827 (−344) CT/TT and rs2251746 (−95) TC were significantly higher in allergic patients (Table 2). The frequency of the rs2427827 (−344) T allele was also significantly higher in allergic patients (Table 3).

**Table 1:** Clinical characteristics of different genotypes of the FcεR1α promoter region in allergic patients (*n =* 52).

Gene	Position	Genotype	Age	Total IgE	Eosinophils	Mite-IgE	ELISPOT	Der p 2-IgE
			Median	Median	Median	Median	Median	Median
**FcεR1α**	**−344**	CC	42.50	97.50	150.00	0.28	281.50	0.31
		CT	21.00	561.00	420.00	11.37	436.00	0.81
		TT	31.00	2370.00	260.00	100.00	780.00	0.97
		*p* ^a^	0.033	**<0.001**	0.013	**<0.001**	**<0.001**	**<0.001**
**FcεR1α**	**−95**	TT	35.00	320.00	160.00	2.20	274.51	0.28
		TC/CC	22.00	1431.25	295.12	49.58	723.56	1.03
		*p* ^a^	0.155	**<0.001**	0.006	**<0.001**	**<0.001**	**<0.001**

Median: The number separating the higher half of a data sample; ELISpot: The number of B cells that could produce IgE after Der p2-induced; Mite-IgE: The specific IgE to dust mite *Dermatophagoides pteronyssinus*; Der p2-IgE: The specific IgE to dust mite *Dermatophagoides pteronyssinus* group 2 allergen-Der p2; and *p* ^a^: ANOVA for Kruskal-Wallis test; Data in bold font: Statistical significance.

**Table 2:** Genotype difference of FcεR1α promoter region between allergic and normal subjects.

FcεR1α SNP No.	Genotype	Allergy (*n =* 52)		Healthy (*n =* 50)		*p*
rs2427827 (−344)	CC	20	(38.5%)	32	(64.0%)	0.0348
	CT	22	(42.3%)	13	(26.0%)	
	TT	10	(19.2%)	5	(10.0%)	
rs2251746 (−95)	TT	45	(86.5%)	48	(94%)	0.0057
	TC/CC	7	(13.5%)	2	(6%)	

*p* for Chi-square test.

**Table 3:** Allele frequency Difference of FcεR1α promoter region between allergic and normal subjects.

FcεR1α SNP No.	Allele	Allergy (Allele *n =* 104)		Healthy (Allele *n =* 100)		*p*
rs2427827 (−344)	C	62	(59.6%)	77	(77%)	0.0279
	T	42	(40.4%)	23	(23%)	
rs2251746 (−95)	T	97	(93.3%)	96	(96%)	0.4011
	C	7	(6.7%)	4	(4%)	

*p* for Chi-square test.

### 2.3. Genetic Effects of SNPs of the FcεR1α Promoter Region on FcεR1α mRNA Expression

**Figure 1:**
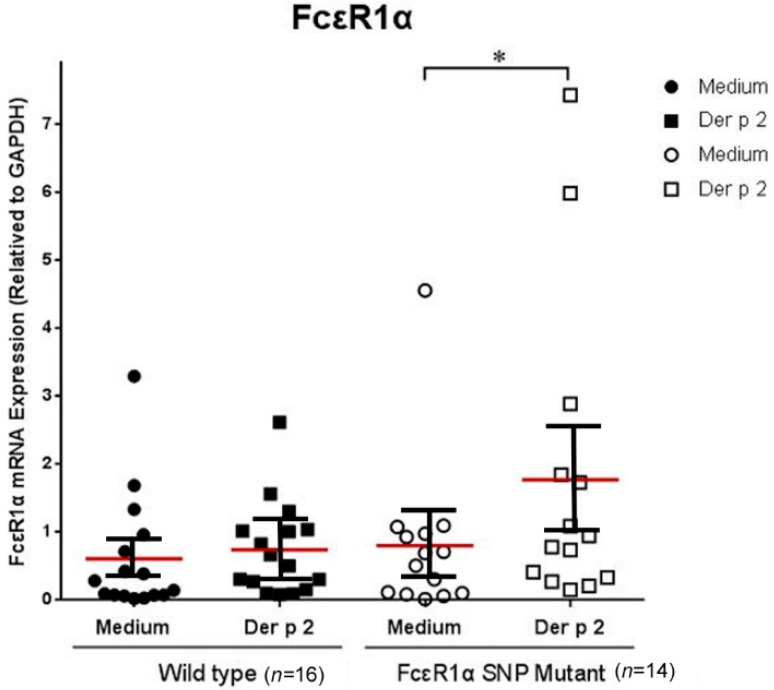
Genetic effect of genotypes rs2251746 (−95, T>C) and rs2427827 (−344, C>T) in the FcεR1α promoter region on FcεR1α mRNA expression. The PBMCs were cultured with or without Der p2 for 48 h, followed by mRNA extraction. Values were presented as mean ± SEM (standard error of mean). There were significantly higher levels of FcεR1α mRNA expression in PBMCs after Der p2 stimulation in subjects with genotypes rs2251746 (−95, T>C) and rs2427827 (−344, C>T) of the FcεR1α promoter region (* *p* < 0.05). Wild-type: Patients with genotypes of rs2251746 (−95TT) and rs2427827 (−344CC) in FcεR1α promoter region; FcεR1α SNP Mutant: Patients with genotypes of rs2251746 (−95TC) and rs2427827 (−344TT/TC) in the FcεR1α promoter region.

The PBMCs derived from allergic subjects with genotypes rs2251746 (−95, T>C) and rs2427827 (−344, C>T) of the FcεR1α promoter region were collected to investigate the effects of Der p2 on FcεR1α mRNA expression. The PBMCs were cultured with or without Der p2 stimulation for 48 h. The cell pellets were collected to extract RNA for mRNA expression. There were significantly higher levels of FcεR1α mRNA expression in the PBMCs after Der p2 stimulation in patients with genotypes rs2251746 (−95, T>C) and rs2427827 (−344, C>T) of the FcεR1α promoter region (Figure 1).

### 2.4. Genetic Effect of SNPs of the FcεR1α Promoter Region on Cε mRNA Expression

**Figure 2:**
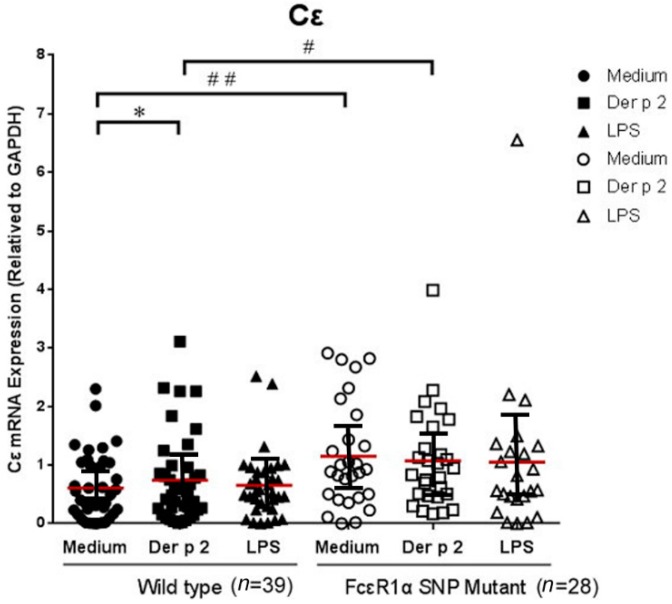
Genetic effect of genotypes rs2251746 (−95, T>C) and rs2427827 (−344, C>T) in the FcεR1α promoter region on Cε mRNA expression. The PBMC were cultured with or without Der p2 for 48 h, followed by mRNA extraction. Values were presented as mean ± SEM. There were significantly higher levels of Cε mRNA expression in the PBMCs of subjects with SNPs of the FcεR1α promoter region, regardless of Der p2 stimulation (* *p* < 0.05); ^#^ *p* < 0.05 when comparisons between the FcεR1α promoter region SNPs (+) and SNPs (−) under Der p 2 (10 μg/mL) induction; ^##^ *p* < 0.05 when comparisons between the FcεR1α promoter region SNPs (+) and SNPs (−) under medium condition. The effects of Der p2 on Cε mRNA expression were not affected by SNPs of the FcεR1α promoter region. Wild-type: Patients with genotypes of rs2251746 (−95TT) and rs2427827 (−344CC) in FcεR1α promoter region. FcεR1α SNP Mutant: Patients with genotypes of rs2251746 (−95TC) and rs2427827 (−344TT/TC) in FcεR1α promoter region.

The PBMCs derived from allergic patients were cultured and analyzed with or without Der p2 and LPS stimulation. The cell pellets were collected for Cε mRNA expression and supernatant were collected for cytokine analysis. There were significantly higher levels of Cε mRNA expression in PBMCs of patients with SNPs of the FcεR1α promoter region compared to those derived from the wild type. However, there was no significantly increased Cε mRNA expression in PBMCs derived from either group after Der p2 stimulation (Figure 2).

### 2.5. Genetic Effects of SNPs of the FcεR1α Promoter Region on Histamine Release

**Figure 3:**
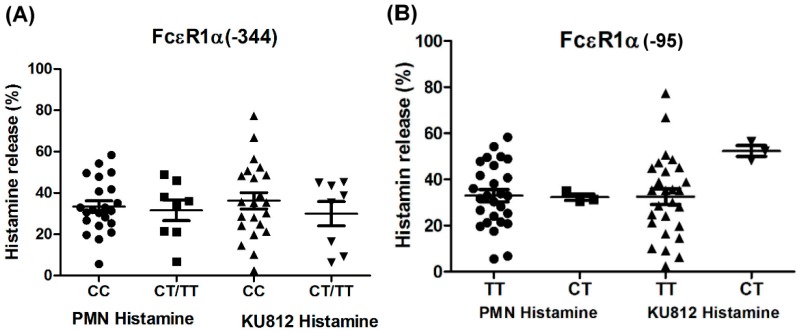
Genetic effect of SNPs of the FcεR1α promoter region on histamine release. The PMNs derived from patients with different FcεR1α promoter SNPs and KU812 cells-presensitized with serum were incubated with Der p2 for 30 min, followed by histamine analysis. Values were presented as mean ± SEM. The results obtained from (**A**) genotype of FcεR1α (−344CC, CT/TT) and (**B**) genotype of FcεR1α (−95TT, CT).

**Figure 4:**
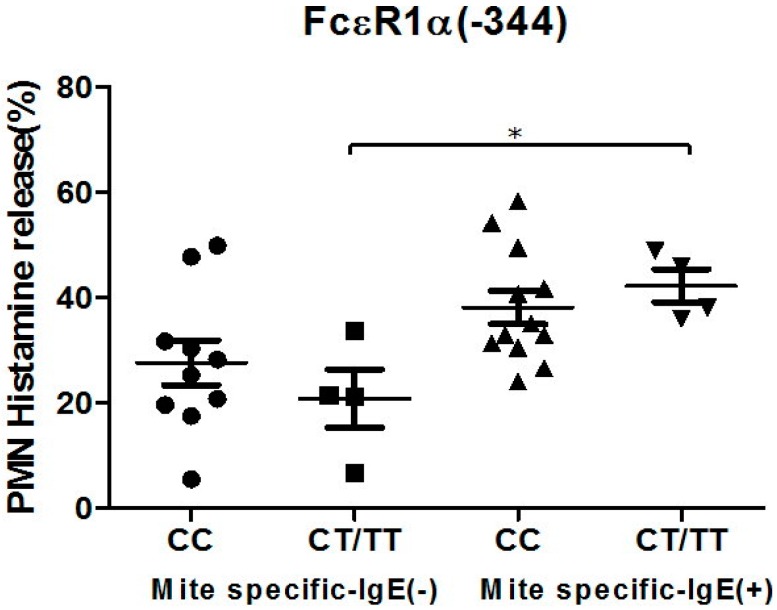
Genetic effect of SNPs of the FcεR1α promoter region (rs2427827, −344) on histamine release. The PMNs derived from patients with different FcεR1α promoter SNPs were incubated with Der p2 for 30 min, followed by histamine analysis. Values were presented as mean ± SEM. PMNs from patients with mite-specific IgE(+) were incubated with Der p2; there was significantly increased histamine release compared to serum derived from mite specific IgE(−); * *p* < 0.05 significance level when comparisons between two groups.

The PMNs with different FcεR1α promoter SNPs were used for the histamine release assay, and the results showed there were no statistically significant differences between the two different FcεR1α promoter genes (Figure 3A,B). Nonetheless, there was a significantly higher level of histamine release from PMNs after incubation with Der p2. When PMNs from patients with mite-specific IgE(+) were incubated with Der p2, there was significantly increased histamine release compared to serum derived from mite specific IgE(−) (Figure 4).

### 2.6. Genetic Effects of SNPs of the FcεR1α Promoter Region on Cytokine Secretion

The PBMCs derived from subjects with genotypes rs2251746 (−95, T>C) and rs2427827 (−344, C>T) of the FcεR1α promoter region were collected for cytokine secretion analysis. There was no significant difference between the two groups except for IL-6 and IL-8 levels before and after Der p2 stimulation. There were increased secretions of IL6 and IL8 in the PBMCs derived from patients with SNPs of the FcεR1α promoter region after Der p2 stimulation. Both IL-6 and IL-8 also increased in subjects with the wild type of FcεR1α promoter region (Table 4). There was no significant difference in cytokine between subjects with genotypes rs2251746 (−95, T>C) and rs2427827 (−344, C>T) of the FcεR1α promoter region in the culture supernatant after Der p2 stimulation (Table 4).

**Table 4:** Comparison of cytokine secretion derived from PBMC after culture with or without Der p 2.

Cytokines	gp	M			rDer p 2			LPS			M *vs.* rDer p2	M *vs.* LPS	rDer p 2 *vs.* LPS
		*n*	Mean	SEM	*n*	Mean	SEM	*n*	Mean	SEM			
IL1-β	1	8	1.80	0.73	8	3.98	1.05	8	59.83	24.80	0.123	0.012	0.012
	2	8	11.07	8.74	8	3.44	1.36	8	23.02	8.24	0.208	0.025	0.161
IL6	1	8	56.69	26.12	8	684.66	255.72	4	9125.61	4810.53	0.012	0.068	0.068
	2	7	61.13	28.44	8	237.54	84.18	6	4183.35	2809.11	0.018	0.116	0.043
IL8	1	8	2379.34	803.78	8	8779.61	2390.56	1	2866.60	1146.64	0.012	0.124	0.035
	2	7	1026.71	279.15	7	5352.49	1448.59	6	13,329.45	4467.66	0.018	0.116	0.028
IL10	1	8	5.00	2.04	8	5.96	1.78	8	127.65	36.64	0.123	0.012	0.012
	2	8	12.82	11.34	8	2.04	0.63	8	53.98	20.13	0.779	0.017	0.093
IL4	1	8	0.62	0.13	8	0.58	0.16	8	0.70	0.14	0.753	0.575	0.735
	2	8	0.85	0.22	8	0.79	0.25	8	0.91	0.36	0.753	0.600	0.684
IFN-γ	1	8	106.14	45.06	8	119.25	61.80	8	476.21	307.18	0.833	0.036	0.069
	2	8	38.10	12.45	8	55.88	34.01	8	127.71	44.87	0.889	0.017	0.025
IL13	1	8	20.39	6.94	8	26.11	11.78	8	14.22	4.89	0.575	0.484	0.263
	2	8	2.65	0.24	8	7.76	5.33	8	7.75	2.89	0.833	0.161	0.012
Eotaxin	1	8	38.12	6.91	8	29.25	6.37	8	36.22	6.18	0.401	0.263	0.866
	2	8	27.60	7.11	8	24.98	7.18	8	30.01	9.76	0.499	1.000	0.600

*p* for Kruskal-Wallis Test; gp1: Patients with genotypes of rs2251746 (−95TC) and rs2427827 (−344TT/TC) in FcεR1α promoter region; gp2: Patients with genotypes of rs2251746 (−95TT) and rs2427827 (−344CC) in FcεR1α promoter region.

### 2.7. Genetic Effect of SNPs of the FcεR1α Promoter Region on IgE Production from B Cells

**Figure 5:**
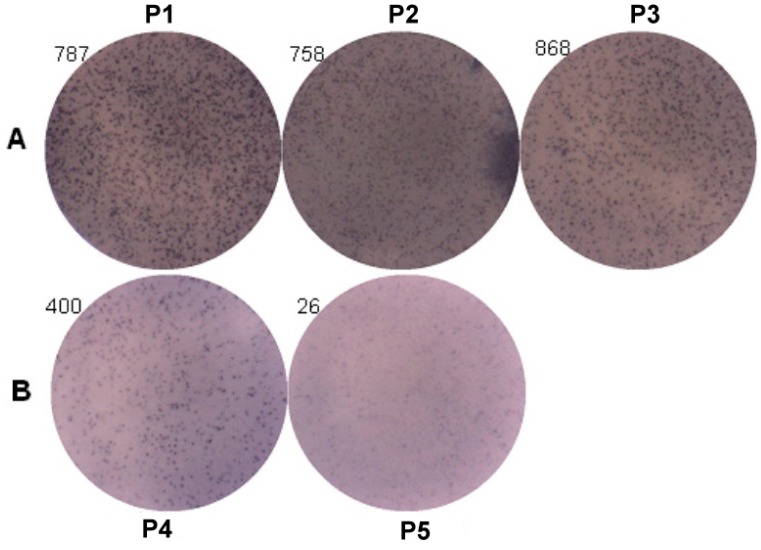
Genetic effect of SNPs of the FcεR1α promoter region on IgE production from B cells. Primary B cells derived from patients with FcεR1α mutant type (**A**) and wild type (**B**) were used for IgE production. Three patients in group A with genotypes (P1: −95TC; P2: −344TT; and P3: −344CT). Two patients in group B with genotypes (P4: −344CC and P5: −95TT). Cells (1 × 10^6^) were treated with IL-4 (30 ng/mL), anti-CD40 (1 μg/mL), IL-21 (25 ng/mL) and rDer p2 (10 μg/mL) for five days. IgE produced B cells were detected by ELISpot assay after treatment.

ELISpot assay was used to analyze the IgE producing B cells (Figure 5). The results of three patients with genotypes (P1: −95TC; P2: −344TT; and P3: −344CT) were 787 spots, 758 spots and 868 spots, and two patients with genotypes (P4: −344CC and P5: −95TT) were 400 spots and 26 spots. The IgE producing B cells were higher in those patients with genotypes of rs2251746 (−95TC) and rs2427827 (−344TT/TC) than those with genotypes of rs2251746 (−95TT) and rs2427827 (−344CC) of FcεR1α promoter.

## 3. Discussion

The associations between SNPs (rs2427827 and rs2251746) in the FcεR1α promoter region and allergic features of Cε expression, histamine release were analyzed, and their effects on leukocyte function compared among the different SNPs were performed in this study. The results showed the two SNPs in the FcεR1α promoter region with CT/TT in rs2427827 and TC in rs2251746 were significantly higher in allergic patients than in non-allergic controls. Patients with the two SNPs (CT/TT in rs2427827 and TC in rs2251746) in the FcεR1α promoter region had high levels of total IgE, mite-specific Der p2-specific IgE and IgE secretion B cells. The mRNA expression of FcεR1α was significantly increased after Der p2 stimulation in PBMCs with the two SNPs. Thus, the results suggest that the SNPs (CT/TT in rs2427827 and TC in rs2251746) in the FcεR1α promoter region are associated with IgE expression, IgE producing B cells, and increase Der p2-induced FcεR1α mRNA expression. These SNPs may be used as a disease marker for IgE-mediated allergic inflammation caused by *Dermatophagoides pteronyssinus*.

Genetic determinants were suspected to influence IgE regulation and IgE levels considerably, and recently genome-wide association studies (GWAS) allowed the identification of the linkage and candidate gene for the total serum IgE levels such as *FCERIA* on human chromosome 1q23 [11]. A large number of studies on links between variations in FcεR1α and allergic diseases such as atopic dermatitis and asthma have been undertaken by many research groups [12]. The *FCERIA* polymorphisms not only drive IgE levels but also influence IgE in association with asthma, suggesting that disease-specific mechanisms in IgE regulation exist [12]. The polymorphism rs2251746 (−95, T/C) in the promoter region of FcεR1α was associated with serum IgE levels, but is not associated with environmental exposures, including indoor endotoxin, day care centre attendance and worm infestation [6]. The association study shows that the genotype polymorphisms of FcεR1α promoter region, rs2427827 and rs2251746, are associated with the phenotype of IgE-related allergic disease and IgE secretion B cells. This discrepancy may be due to the different SNPs analyzed or to ethnic variation. Similar results have been reported by Park *et al.* [13].

The high affinity receptor for IgE, FcεRI, is expressed on several different types of cells such as basophils/mast cells, epidermal Langerhans cells, dermal dendritic cells, monocytes, and eosinophils. Among these cells, the receptor on basophils/mast cells is composed of three different subunits that form αβγ_2_ heterotetramer, but on the other cells only two subunits forming αγ_2_ heterotrimer. The structure and function of the *FcεR1A* gene, encoding for α-subunit of FcεRI (FcεRIα), plays an important role in the pathogenesis of allergic diseases. It has been reported that the FcεR1α receptor gene play important roles in IgE synthesis and IgE-mediated response [14]. The basophils and monocytes are the rich FcεR1-expressing cells that circulate in the peripherial blood of allergic individuals. The cell-type specific expression of the α-subunit coincides well with the FcεRI-expressing cell-type specificity. Involvement of the α-subunit in FcεR1-mediated allergic reaction has been demonstrated by the absence of allergic reactions in α-chain-deficient mice. Several studies described a genetic association between *FcεR1A* variants and total serum IgE levels in allergic subjects. Similar findings of significantly higher levels of Cε mRNA expression in PBMCs derived from subjects of genotypes with −95C and −344T alleles of the FcεR1α promoter region compared to those derived from genotypes with −95T and −344C alleles have been observed. These results suggest that the two SNPs of the FcεR1α promoter region not only affect FcεR1α expression but also Cε expression.

The functional investigation of the FcεR1α promoter showed the −66T/C polymorphism affecting the transcription activity GATA-1-binding affinity, which is one of the determinants for allergic diseases [15]. The −344C/T polymorphism of the FcεR1α promoter is significantly associated with total serum IgE concentrations and a higher rate of patients with aspirin-intolerant chronic urticaria, it suggests the −344T allele exhibits significantly higher promoter activity when compared with the −344C allele [16]. The functional assay shows the α-chain of the FcεR1 promoter carrying −315T (minor allele) possesses significantly higher transcriptional activity than that of −315C (major allele). It demonstrates that a −315 SNP significantly affects human FcεRI α-chain promoter activity and expression level of FcεRI on basophils by binding transcription factor Sp1 to the SNP site [17]. In this study of functional investigation, the expression of Cε from subjects with both wild type and SNP mutation is not affected by Der p2, suggesting that this promoter gene is not susceptible to Der p2. The SNPs are relevant to Der p2-induced FcεR1α mRNA expression but not Cε expression.

The IgE mediated immune responses are best known for their involvement in allergies, and genetic determinants are suspected to influence IgE regulation and IgE levels considerably. Recently GWAS have provided the power to identify genetic determinants for serum IgE levels such as 1q23 (*FCER1A*), 5q31 (*RAD50*, *IL13*, *IL4*), 12q13 (*STAT6*), 6p21.3 (*HLA-DRB1*) and 16p12 (*IL4R*, *IL21R*), suggesting that genetic variants in these loci may influence IgE regulation [11]. The SNPs within regulatory elements of *FCERIA* have functional associations, which were reported and replicated in several research groups. These associations do not confer susceptibility to allergic diseases, but rather modulate serum concentrations of IgE. *FCERIA* is a good candidate in allergic diseases, and appears to participate in system regulation of IgE levels [18]. In this study, the Cε mRNA expression levels were significantly higher in subjects with FcεR1α promoter SNPs. In the Cε expression study, there are significantly higher levels of Cε mRNA expression in PBMCs derived from subjects with SNPs of the FcεR1α promoter region compared to those derived from the wild type, suggesting that the Cε expression related to IgE synthesis may be affected by functional regulation of the FcεR1α promoter SNPs. Although FcεR1α mRNA expression can be up-regulated by Der p2, Cε mRNA expression cannot be further up-regulated. This indicates that Cε expression in PBMCs can be triggered by antigens other than Der p2. The mechanisms involved warrant further investigation.

Basophils in PMNs have been reported to express FcεR1α and be activated though cross-linking of antigen-specific IgE by antigen [19]. In this study, histamine released from PMN has also been observed in incubation with Der p2 and dust mite-specific IgE positive serum. However, the levels of histamine released from Der p2-triggered PMNs from patients with SNPs of the FcεR1α promoter gene are similar to those of PMNs derived from patients without mutation. These findings suggest that histamine release from PMNs is not affected by FcεR1α genetic polymorphisms. Since histamine release from PMNs is affected by pre-bound IgE and specific antigen, this may be because pre-bound IgE and antigen are not specific to Der p2. However, the detailed mechanisms cannot be clarified until the allergen specific IgE on the PMNs is identified, especially since various inflammatory cells aside from PMNs of basophils and eosinophil are recruited to the airways after allergen challenge.

Many cells function as APCs to initiate or enhance Th2 responses directly or indirectly through cytokines [20]. Culture of PBMCs in the presence of allergen can result in inflammatory cytokine release and Th2 cell differentiation [20]. This study reveals that the secretions of IL-6 and IL-8 from PBMCs of patients with or without SNPs of the FcεR1α promoter region are significantly increased after Der p2 stimulation (Table 4). However, this study fails to determine the difference in cytokine secretion between SNPs of the FcεR1α promoter region and the wild type (Table 5). It suggests that there is no direct association between SNPs (rs2427827 and rs2251746) in the FcεR1α promoter region and cytokine production. The sections of inflammatory cytokine (IL-6) and chemokine (IL-8) were significantly associated with the allergen Der p2 exposure. The SNPs (CT/TT in rs2427827 and TC in rs2251746) in the FcεR1α promoter region were strongly associated with IgE-mediated allergic responses. Taken together, these results indicate that Der p2-stimulated cytokine release from PMBCs is not affected by SNPs of the FcεR1α promoter region (rs2427827 and rs2251746). Similar findings have been reported that house dust mite (HDM) exposure does not result in a classical TH2-driven response, but was more representative of noneosinophilic asthma unless rhinovirus is used as an adjuvant [21]. The −66T/C polymorphism of the FcεR1α promoter showed a significant portion of nonallergic individuals with heterozygous TC genotype, while most of allergic individual have homozygous TT genotype in the Japanese population [15]. However, the TC genotype and minor allele C of rs2251746 (−95) were observed to have a higher portion of allergic patients with higher total IgE and mite-IgE in this study; these results may be attributed to the different populations, and whether the sample size was large enough. The major limitation of this study was too few cases in the genetic association study; so we refer to it as an exploratory pilot study while more cases are included in further research. In conclusion, the results of this exploratory pilot study showed SNPs (rs2427827 and rs2251746) in the FcεR1α promoter region are associated with the phenotype of IgE-mediated allergic diseases, such as total IgE, Der p2-specific IgE, IgE producing B cells, Der p2-induced FcεR1α mRNA expression and Cε expression. These findings suggest that SNPs may play an important role in the IgE-mediated allergic inflammation caused by *Dermatophagoides pteronyssinus*.

**Table 5:** Comparison of cytokine secretion derived from PBMC between SNPs of FcεR1α promoter region and wild type.

*p* for Mann-Whitney Test	gp1 *vs.* gp2		
	M	rDer p 2	LPS
IL6	0.867	0.195	0.476
IL8	0.336	0.281	0.857
IL10	0.959	0.065	0.161
Eotaxin	0.382	0.798	0.234
IFN-γ	0.234	0.574	0.798
IL1-β	0.959	0.721	0.328
IL13	0.234	0.234	0.382
IL4	0.645	0.645	0.645
TNF-α	0.721	0.161	0.721

*p* for Kruskal-Wallis Test; gp1: Patients with genotypes of rs2251746 (−95TC) and rs2427827 (−344TT/TC) in FcεR1α promoter region; gp2: Patients with genotypes of rs2251746 (−95TT) and rs2427827 (−344CC) in FcεR1α promoter region.

## 4. Material and Methods

### 4.1. Study Subjects

The Institutional Review Board of Taichung Veterans General Hospital approved the study protocol (File number CF12010 and CF12009) and each participant provided written informed consent before enrollment. Sixty-seven patients with airway allergy and 50 non-allergic subjects who consulted at the Allergy Clinic of Taichung-Veterans General Hospital were recruited. There were 36 males and 31 females in these allergic patients. The age ranged from 8 to 48 years old, with the mean and standard deviation as 27 ± 15.6. Patients with airway allergy were defined as having a history of recurrent nasal stuffiness, sneezing, and/or asthma. Serum total IgE and mite specific IgE were measured by using the UniCAP system (Thermo Fisher Scientific, Uppsala, Sweden) as background value. Cytokine and histamine release from leukocytes derived from different genetic polymorphisms were also examined.

### 4.2. SNP Genotyping Analysis

Blood samples (5 mL) were collected with EDTA syringes and DNA from the buffy coat was purified using a Genomic DNA Mini kit (Geneaid, Taoyuan, Taiwan). A set of primers was designed for the amplification 684 base pair PCR product based on FceR1α promoter region. The forward and reverse primer sequences were 5'-AAGAAAAGCGTTGGTAGCTCTGGTG-3' and 5'-ATCTTCTTCATGGACTCCTGGTGC-3', respectively. The genotype and frequency of the FcεR1α promoter region with CT and TT in rs2427827 (−344) and TC in rs2251746 (−95) between allergic patients (*n* = 52) and healthy controls (*n* = 50) were compared. Two SNPs rs2427827 (−344) and rs2251746 (−95) in the promoter region were identified by direct sequencing of the PCR products of genomic DNA.

The FcεR1β and FcεR1γ genotypes were determined using a TaqMan real-time polymerase chain reaction (PCR) (TaqMan SNP Genotyping Assays; Life Technologies, Foster City, CA, USA) in a StepOnePlus™ Real-Time PCR System (Applied Biosystems, Foster City, CA, USA). The Assay IDs of rs1441586 and rs11587213 were C_1842226_10 and C_27848237_10, respectively. Negative controls were included in each PCR to avoid contamination. Detection of the probe signal with two ABI fluorescent dyes (FAM™-Allele 1 and VIC^®^-Allele 2) was conducted using 96-well plates in an ABI Prism 7900 Real-Time PCR system (Applied Biosystems). Duplicate samples and negative controls were included to ensure genotyping accuracy.

### 4.3. PBMC Culture and Cytokine Analysis

Human PBMCs were prepared by density centrifugation Ficoll-paque^™^ PLUS (GE Healthcare, Uppsala, Sweden). The cells were stimulated with or without Der p2 (Group 2 allergen of *Dermatophagoides pteronyssinus*; Indoor Biotechnologies Ltd., Cardiff, UK), or LPS (*Escherichia coli* strain 055:B5; Sigma-Aldrich, St. Louis, MO, USA) for 3 days in RPMI-1640 medium containing 10% heat inactivated FBS and 1% streptomycin/penicillin in a humidified 5% CO_2_ atmosphere. After the PBMCs cultured with or without Der p2 stimulation, the cell pellets were collected to extract RNA for mRNA expression. The cell culture supernatant were collected and evaluated for cytokine concentrations using commercially available Luminex MAP^®^ kits according to the manufacturers’ directions. Correlations between data sets were evaluated using Pearson’s correlation coefficient (*r*).

### 4.4. B Cell Culture with ELISpot Assay

Human PBMCs were prepared by density centrifugation Ficoll-paque™ PLUS. B cells were then purified by negative selection with a B cell isolation kits. Non-B cells were indirectly magnetically labeled by using a cocktail of biotin-conjugated antibodies against CD2, CD14, CD16, CD36, CD43, CD235a (Glycophorin A), and Anti-Biotin MicroBeads (Miltenyi Biotec., Bergisch Gladbach, Germany). Isolation of highly pure B cells is achieved by depletion of magnetically labeled cells. Purified B cells were cultured with Der p2 (10 μg/mL) in the presence of IL-4 (30 ng/mL), IL-21 (25 ng/mL) and anti-CD40 (1 μg/mL) for 5 days in the CO_2_ incubator. ELISpot assays were performed utilizing Mabtech™ ELISpot plus Human IgE kits (MABTECH AB, Nacka Strand, Sweden). The number of spot-forming cells in each well was automatically counted with a Cellular Technology Limited (CTL)-ImmunoSpot Analyzer (Cellular Technology Ltd., Shaker Heights, OH, USA)

### 4.5. Reverse Transcription-Polymerase Chain Reaction (RT-PCR)

The PBMCs derived from allergic patients were cultured with or without Der p2 or LPS for 3 days, and the cell pellets were then collected to extract RNA for Cε mRNA expression and supernatant for cytokine analysis. Total RNA from cells was extracted using the RNeasy mini kit (Qiagen, Valencia, CA, USA). The first standard cDNA was synthesized by the RevertAid M-MuLV reverse transcriptase (Thermo Fisher Scientific, Uppsala, Sweden) according to the manufacturer’s protocol. The cDNA then served as a template in a PCR using the G-Storm PCR machine (GS4822, Somerton Biotechnology Centre, Catcombe, UK). The IgE synthesis from B-cells carries out from the IgM to isotype IgG, which mRNA expression from Cμ gene to Cε gene. Detection of excision circular transcripts from direct Cμ gene to Cε switching events was performed using a forward primer in Iε (5'-CGTCTTCCCCTTGACCCGCTGCTG-3') together with a reverse primer in Cμ (5'-CACGTCCATGACCTGCCCGTCCTC-3'). The amplification cycles were as follows: 94 °C for 30 s, 60 °C for 30 s, and 72 °C for 60 s. The PCR products were then subjected to electrophoresis on a 2% agarose gel after 30 cycles and the electrophoresis products were visualized by ethidium bromide staining. The mRNA of GAPDH was used to control sample integrity and loading.

### 4.6. Basophil Histamine Release Assay

Sera from 30 patients with airway allergy or non-allergic controls were collected for basophil histamine release assay. The patients were instructed to avoid the use of systemic anti-allergenic drugs (*i.e.*, anti-histamines and systemic corticosteroids) for at least 24 h before blood sampling.

Histamine release assay was analyzed after incubation with Der p2 for 30 min. The basophil cell line KU812 was used as control. The polymorphonuclear cells (PMNs) were isolated from the blood of allergic patients by centrifugation of the leukocytes on Ficoll-paque™ PLUS. KU812 cells (human basophilic leukemia cell lines, purchased from Riken Cell Bank, Tsukuba, Japan) were re-suspended in medium RPMI-1640 by adjusting to 1 × 10^6^ cells/mL using trypan blue assessment. Passive sensitization of KU812 cells with sera from patients or controls (1/5 volume of subject sera) was performed for 4 h at 37 °C.

After incubation with sera, the sensitized KU812 cells or PMNs from allergic patients were then incubated with 10 μg/mL of Der p2 for 30 min at 37 °C and with 1 μg/mL of A23187 (Sigma–Aldrich Co., St. Louis, MO, USA) as total release histamine. The supernatant was collected and reacted with *O*-phthalaldehyde (OPA, 5 mM) for 7 min. The reaction was stopped by adding H_2_SO_4_ (0.04 M). The histamine released into the supernatant was measured by a fluorescence spectrophotometer. The percentage of histamine release was calculated using the following formula:

(Stimulated released histamine − Spontaneous released histamine)/(Total released histamine − Spontaneous released histamine) × 100%

### 4.7. Statistical Analysis

All statistical analyses were performed using SPSS software (version 22; SPSS Inc., Chicago, IL, USA). The SPSS Sample Power 2.0 was used for power calculation analysis. Values were presented as mean ± standard error of mean (SEM). All analyses were performed by analysis of variance (ANOVA) with non-parametric testing, followed by Spearman’s coefficient and Kruskal-Wallis Test. Statistical significance was set at *p* < 0.05.

## 5. Conclusions

SNPs of the FcεR1α promoter region (rs2427827 and rs2251746) are associated with the phenotype of IgE-mediated allergic diseases, including total IgE, Der p 2-specific IgE and IgE producing B cells. The SNPs of the FcεR1α promoter regions rs2427827 and rs2251746 are associated with Der p2-induced FcεR1α mRNA expression and high levels of Cε expression. These findings suggest that SNPs may play an important role in the allergic inflammation caused by *Dermatophagoides pteronyssinus*.

## Supplementary Materials

Supplementary materials can be found at http://www.mdpi.com/1422-0067/16/05/9504/s1.
