# Supplementary material for: An Exploratory Pilot Study of Genetic Marker for IgE-Mediated Allergic Diseases with Expressions of FcεR1α and Cε
Source: Int J Mol Sci. 2015 Apr 27;16(5):9504–19. doi: 10.3390/ijms16059504 (PMC4463601; doi:10.3390/ijms16059504)
Supplement: Supplementary file 1 [file ijms-16-09504-s001.pdf]

Supplementary Information

Table S1. Comparison of clinical characteristics between allergic and healthy subjects.

Group (Number)	Gender	Age	Total IgE	Eosinophils	Mite-IgE	ELISPOT	Der p2-IgE
	(Male:Female)	Median	Median	Median	Median	Median	Median
**Allergy (52)**	28:24	28	851.51	206.32	2.86	521.63	0.64
**Healthy (50)**	26:24	26	245.73	155.63	2.15	ND	0.22
p	0.78	0.72	**<0.05**	0.055	**<0.05**	–	**<0.05**

Median: The number separating the higher half of a data sample; ELISPOT: The number of B-cells which could produce IgE after Der p2-induced; Mite-IgE: The specific IgE to dust mite *Dermatophagoides pteronyssinus*; Der p2-IgE: The specific IgE to dust mite *Dermatophagoides pteronyssinus* group 2 allergen-Der p2; ND: non-detected; *p* for Chi-square test; $p < 0.05$ significance level; and "–": It cannot be compared.
